# Towards an integrated food safety surveillance system: a simulation study to explore the potential of combining genomic and epidemiological metadata

**DOI:** 10.1098/rsos.160721

**Published:** 2017-03-29

**Authors:** A. A. Hill, M. Crotta, B. Wall, L. Good, S. J. O'Brien, J. Guitian

**Affiliations:** 1 CORDA, BAE Systems, Farnborough, UK; 2 Royal Veterinary College, University of London, London, UK; 3 NIHR Health Protection Research Unit in Gastrointestinal Infections, UK

**Keywords:** surveillance, food safety, systems analysis

## Abstract

Foodborne infection is a result of exposure to complex, dynamic food systems. The efficiency of foodborne infection is driven by ongoing shifts in genetic machinery. Next-generation sequencing technologies can provide high-fidelity data about the genetics of a pathogen. However, food safety surveillance systems do not currently provide similar high-fidelity epidemiological metadata to associate with genetic data. As a consequence, it is rarely possible to transform genetic data into actionable knowledge that can be used to genuinely inform risk assessment or prevent outbreaks. Big data approaches are touted as a revolution in decision support, and pose a potentially attractive method for closing the gap between the fidelity of genetic and epidemiological metadata for food safety surveillance. We therefore developed a simple food chain model to investigate the potential benefits of combining ‘big’ data sources, including both genetic and high-fidelity epidemiological metadata. Our results suggest that, as for any surveillance system, the collected data must be relevant and characterize the important dynamics of a system if we are to properly understand risk: this suggests the need to carefully consider data curation, rather than the more ambitious claims of big data proponents that unstructured and unrelated data sources can be combined to generate consistent insight. Of interest is that the biggest influencers of foodborne infection risk were contamination load and processing temperature, not genotype. This suggests that understanding food chain dynamics would probably more effectively generate insight into foodborne risk than prescribing the hazard in ever more detail in terms of genotype.

## 1. Introduction

The most important enteric disease burdens worldwide are caused by microbiological hazards such as *Salmonella* spp. and *Campylobacter* spp., which can cause human infection via both food and environmental routes. In the UK, good progress has been made in reducing the number of *Salmonella* cases over the past couple of decades, although *Campylobacter* cases remain stubbornly high [1,2]. The large burden of disease reflects the exposure of people to a complex food chain of ever-changing trade routes, production processes and consumption patterns that interact with the ongoing evolution of enteric pathogens. The efficiency of zoonotic infection, and the virulence of that infection, will be driven by the constant shift in the genetic machinery that power bacterial and viral pathogens. Therefore, both the food chain and genotype should be considered when assessing zoonotic risk. To date, research into the combination of food chain data with genetic pathogen data has been scarce, indeed most papers referencing genetic epidemiology simply state the case for the use of genetic information in epidemiology, rather than suggesting how to combine data sources [3–5]. In this paper, we present a hypothetical, quantitative case study, focusing specifically on meatborne infection, to highlight the value of combining both genotypic and epidemiological metadata.

Traditional identification or typing systems based on phenotype (i.e. serotype, biotype, phage-type or antibiogram), have been used for many years. Since the initial use of plasmid fingerprinting in the 1980s [6], the molecular characterization of bacterial isolates revolutionized the ability to differentiate bacterial subtypes and has become an essential component for epidemiological investigation of infectious disease, enhanced surveillance and detection and source attribution of outbreaks caused by foodborne pathogens [7,8]. Whole-genome sequencing (WGS) is part of a revolution involving the so-called next-generation sequencing (NGS) technologies, a set of methods that can reveal the entire genome of an organism. In-field applications of WGS have garnered success in multiple contexts in recent years: from transmission studies and outbreak tracking of pathogens in hospitals [9–11] to traceback investigations and source-attribution in foodborne outbreaks at both national and multinational levels [12–14].

In a recent review by the European Food Safety Authority (EFSA) [15,16], it was recognized that NGS and WGS technologies are not currently used to their full potential with regards to risk assessment and epidemiological insight. The reasons for this include, but are not limited to: (i) the construction of the necessary infrastructure to deal with the volume of sequencing data generated, (ii) deciding which metrics are useful in evaluating risk, and (iii) what metadata should be collected with the genomic data to set the context of the system in which the WGS sample was taken. Each of these reasons alone represents a formidable challenge in turning genomic data into actionable knowledge that can be used to improve surveillance and control. EFSA notes that ‘at present, prototype databases cannot be used for surveillance purposes since they are not widely linked to epidemiological data’. Put simply, the high fidelity and resolution of NGS technologies is not matched by the complementary metadata.

In parallel to the explosion in available genetic sequencing data, another revolutionary technology is the networking of systems and sensors and the generation of big data (colloquially known as the internet of things). However, this revolution in ‘big data’ is largely unexplored in food safety research. Networked sensors can provide real-time, centrally collated data on the performance of production systems as is used in various industries, from energy to car manufacturing. In addition, radio frequency identification (RFID) tagging provides the ability to track and trace individual items, as well as providing a wealth of data for individual products. The application of these technologies could potentially revolutionize food production efficiency and quality, and indeed several trials of tracking and tracing using RFID have been published [17–19]. These technologies provide the potential to generate a similarly high-discriminatory power for epidemiological data as for NGS technologies, which we believe is a key missing link in translating WGS data into actionable knowledge.

A cautionary note must be struck when discussing the potential of big data: it is sometimes promoted as a panacea to almost all analytical problems, but we must always be mindful of the need to construct decision support evidence (using big data or otherwise) with analytical and statistical rigour. Just because there is a large dataset to work with does not mean it is representative of the study population or contains all relevant contextual metadata. Our working hypothesis is therefore that we must have high fidelity but *relevant* epidemiological metadata if we are to genuinely transform genomic data into actionable knowledge that can assist policy-makers to make informed decisions about food safety risk. There will still be limits on the insight that any feasible surveillance system can generate, especially as most food chains are complex and subject to a large volume of noise in the system. Therefore, our aim in this paper is to begin to explore our hypothesis by using a simple food chain model to investigate how detailed epidemiological data may fit into a food safety surveillance system, and ultimately to begin to assess the potential benefits that such data may bring for public health risk assessment.

## 2. Method for investigating the combination of genomic and epidemiological metadata

### 2.1 A brief review of food safety surveillance and predictive analytics

Broadly speaking there are two main objectives of a food safety surveillance system that monitors the incidence of foodborne disease: (i) to inform policies that reduce the number of cases over time, and (ii) to identify and control foodborne outbreaks as and when they occur. These two objectives are not mutually exclusive, but differentiate the emergency response and the longer-term ongoing refinement of any national surveillance system.

WGS and NGS technologies provide unprecedented discriminatory detail, but there remains a question over how best to apply this knowledge within a risk-based surveillance system that should primarily be geared to preventing human exposure to large doses in food items. Dividing up total burdens of a pathogen into ever-more discrete subtypes may increase the sensitivity of detecting outbreaks, but alone it is unlikely to be especially helpful for a general surveillance framework that aims to reduce overall foodborne illness. However, being able to identify, evaluate and prioritize novel and higher-risk pathogens/genes within the diverse range of foodborne organisms would be a key contributing factor to mitigate along the food chain and to prevent the (re-)emergence of potentially dangerous pathogens.

The advances in computing power and big data generation within the past decade or so means that it is now possible for food safety scientists and analysts to quickly, and relatively cheaply, analyse huge datasets. The great promise of this increase in analytical capability is that analysts may start to better inform decision-makers by interrogating large, unrelated databases for trends and patterns that can then be used to forecast what will happen in near real-time. From a food safety point of view, if we had relevant big data we could theoretically move from simply reporting the past (weekly or monthly summaries of how many cases there have been), to describing in more detail what is happening now or in the recent past (arguably where we are with the current suite of early detection algorithms), and eventually into the realm of being able to forecast what might happen or even to prevent an outbreak occurring (predictive analytics). Simply put, such analytical capability would enable us to move from reacting to outbreaks already underway, to identifying higher-risk scenarios and militating against them ahead of time.

Again, we must strike a cautionary note. An obvious tension between the promise and reality of predictive analytics is that these large, potentially unrelated, databases still have to be relevant to the decision in hand. Simply combining available, disparate epidemiological data sources with genetic data and/or without specific risk questions in mind, is unlikely to result in systematic advances in knowledge. Our main interest therefore lies in understanding the decision-support benefit of combining *relevant* genetic sequencing datasets with *relevant* ‘big’ epidemiological data. Without this linkage we are missing the detailed contextual information that allows us to assess risk at even a basic level, yet alone to being able to forecast with any confidence. We are at the beginning of truly acquiring such capability, but arguably the technologies to provide the big data required do already exist: it is a matter of understanding which big data will provide the most added value, and how to generate and robustly analyse these datasets.

### 2.2 Analysis framework

This preliminary study was aimed at understanding the extra value that can be gained by combining genetic data with epidemiological metadata. All food chains, but especially for meat, are inherently long, complex and dynamic. A serving of meat on a plate has involved the growing, transportation and processing of feed ingredients, the breeding, rearing, slaughter and processing of meat animals, to the distribution, storage and preparation of the meat to consume. Such a long food chain contains many and disparate ingredient sources and processes, with multiple opportunities for contamination of the chain with pathogens and for human/system error to fail to reduce any pathogen contamination to an acceptable level. It is therefore extremely difficult to collect sufficiently robust, routine surveillance data that can be used to separate the ‘noise’ of day-to-day production from the ‘signal’ of malfunctions or gross contamination events that lead to foodborne infection or wider outbreaks. For example, the most detailed information on a foodborne outbreak is likely to come from the sampling and questionnaires of human cases once an outbreak has been identified, potentially long after any evidence for the cause of the outbreak has disappeared or been overwhelmed by noise. Therefore, we rarely have a complete understanding of the cause of an outbreak or ongoing infections, and hence it is also extremely difficult to decide what available data would have been useful in source attribution (or what other data could have been useful if they were available).

We therefore developed a hypothetical food chain simulation, so that we can know, with absolute precision: (i) the microbiological status of each product throughout the food chain, (ii) the performance of the equipment being used at the time of the processing of the product, and (iii) whether or not it caused a human infection. In this way, we can compare the performance of a surveillance system (with varying levels of incomplete knowledge) against the ‘gold standard’ of our hypothetical, but completely known, simulation. We then investigated what observations we could make about the simulated baseline dataset by assuming different surveillance systems, each reliant on progressively more detailed epidemiological data. The advantage of this hypothetical approach is that we are able to explore the capabilities and limitations of the methodology, and what data are most beneficial to collect, rather than be constrained by the scarcity and quality of the data actually available.

**Figure 1.**
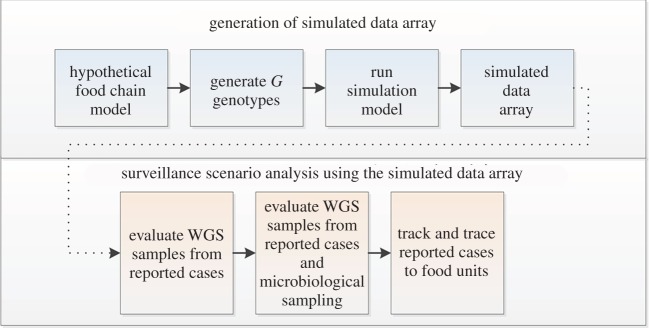
Chronology of the analytical framework. A simulated data array, containing relevant epidemiological and genomic data, was generated from a simple food chain model. We then assumed various rows and columns of the data array could be observed with progressively more advanced surveillance systems. We then interrogated the subsets of the data array (representing different surveillance strategies) to determine the relationships and trends observable by first accessing detailed WGS data alone, and then adding the ability to collect detailed epidemiological data via tracking and tracing systems.

The chronology of the analytical framework was to generate a simulated data array from which to test progressively more advanced surveillance systems (figure 1). The main steps were to:

1. generate a simulated dataset from the hypothetical food chain model. The unit of interest is a food unit (unit=serving). The output of this model is whether the food unit results in human infection from one or more of *G* genotypes;
2. visualize the simulated dataset and understand the relationships between individual parameters and the output. Use these relationships to guide understanding of the outputs of the more advanced analyses; and
3. assume various surveillance strategies based on access to progressively more WGS and/or epidemiological data. Extract a subset of the baseline dataset to replicate the assumed surveillance strategy and the observations that surveillance strategy would allow. For example, current surveillance systems that really only capture WGS data at the human end of the food chain would not allow observation of the variation in WGS during processing of food units, whereas a surveillance system collecting WGS through microbiological sampling criteria would allow such information to be captured and analysed.

### 2.3 Generating baseline simulated dataset of human infections and related food unit parameters

#### 2.3.1 Hypothetical food chain model framework

An amenable food chain model should be one that describes in sufficient complexity the performance of industry equipment as well as the inactivation/cross-contamination of zoonotic pathogens (such that we can model the inclusion of both WGS and epidemiological big data). A model with these characteristics was previously developed for an EFSA risk assessment of *Salmonella* in pigs [20]. In this risk assessment, pigs and their carcasses/food products were modelled individually across the entire production chain, with a high level of focus on the performance of processing steps, for example scalding and the evisceration process of the pig carcass [21].

We chose two specific stages of the model, scalding and evisceration, as being representative of common food processing practices [21]. Scalding is an example of a hot water bath used in various scenarios during food manufacture, for example, to soften hair/feathers on animal carcasses before they are removed, or to partially pasteurize a product. This process is a classic example of both thermal inactivation and washing of a product to produce a critical control point (CCP). On the other hand, inappropriately conducted, scalding is a hazard that can increase the risk of cross-contamination. The evisceration of a carcass can be a gross contamination point if precautions are not taken to minimize the leakage of faecal material from the viscera to the carcass.

**Figure 2.**
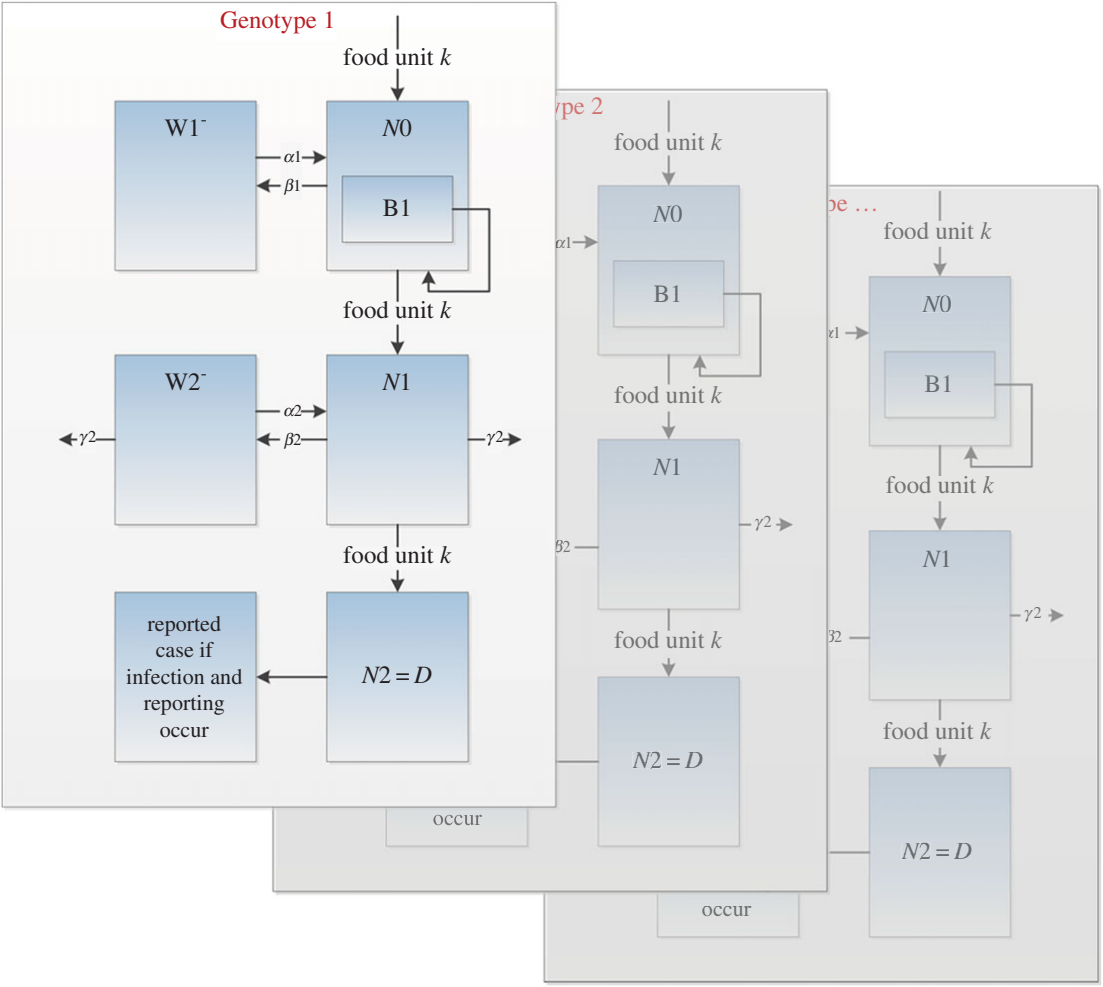
Overall framework for hypothetical model. The processing steps are generalized from [21]. A unit *k* enters process 1 (akin to evisceration) with various burdens of contamination from different genotypes (the model process is applied to each genotype independently, as shown by the faded out replications for Genotype 2 and up). The number of each genotype on each unit is tracked as various cross-contamination and inactivation processes spread and reduce loads across the two processes (more detail on the notation used can be found in appendix A). A case of genotype *g* may then occur according to a function of *D*_*k*,*g*_, the dose of genotype *g* ingested from unit *k*.

We generalized these two processes to represent a variety of processing conditions that a food safety surveillance system could potentially capture. We swapped the order of the two processes around, so that we have a gross contamination event first, followed by a CCP stage. For simplicity, we assume that the unit being processed is a typical serving of meat, and the dose received by the consumer is the contamination present at the end of the second processing step. We outline the overall model framework in figure 2.

**Figure 3.**
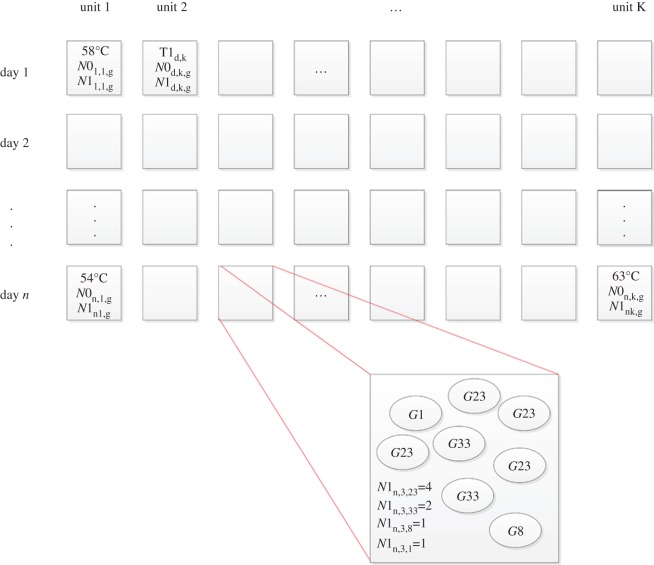
Schematic of stored data array, using the scalding process as an example. The day and position of the unit processed form the first two dimensions of the stored data array, where the temperature of the scalding bath is stored at each position. The exploded diagram shows a number of bacteria of varying genotype (*g*=1.*G*) on unit (*d*,*k*). We store the number of each genotype sub-population *g* on the unit at the end of scalding (*N*1_*d*,*k*,*g*_), creating a three-dimensional array of contamination loads for each genotype on a unit *k* processed on day *d*.

A more complete description of the mathematical model is given in appendix A, but here we concern ourselves with the general principles of the model dynamics, and how we have modified the original model by Swart *et al.* [21] to include varying genotype and phenotype. Briefly, the model tracks over time the exterior contamination of individual food units and the environment (e.g. the scalding water or the evisceration equipment) with a hypothetical bacterial pathogen, *X*. The three main mechanisms of change in the bacterial population modelled within the processes are thermal inactivation, detachment of bacteria through washing, and cross-contamination of equipment/water and other food units. We modelled *G* populations of independent genotypes *g* (with varying phenotypic responses to temperature, attachment efficiency, etc.), while also constructing the ability to record key information along the way. A simple graphical explanation of the data collected is given in figure 3, using the scalding process as an example.

We calculated the probability of infection, *P*_*k*,*g*_, using a Beta-Binomal dose–response model [22], and we assumed the likelihood of laboratory reporting of the case (along with a WGS sample of the particular genotype/s that caused infection) was proportional to a virulence factor, *v*_*g*_. We assumed that each sub-population present on a unit at consumption acts independently to cause infection; hence a person may be infected with several genotypes in any one exposure event (we assumed WGS surveillance would distinguish this multiple infection scenario and assign a case to each of the one or more genotypes causing infection within a single case). The overall likelihood of a laboratory report given consumption of unit *k*, *L*_*k*,*g*_, is given by

2.1 $$L_{k,g}∼[1-{\left(1-pm_{g}\right)}^{D_{k,g}}]v_{g},$$

where ∼ means ‘sampled from’. For each unit *k* on day *d*, we determined whether a case was reported or not using *R*_*k*,*g*,*d*_∼*B*(1,*L*_*k*,*g*_) for each day *d* (where *B* represents a binomial distribution). We summed the number of total laboratory reports over a week's worth of production/consumption (assuming that the unit consumed is fresh produce consumed near the processing date), that is in one week the total number of cases would be given by $\sum_{d}^{7}\sum_{g}\sum_{k}R_{k,g}$. This then replicated, in a simple fashion, the manner and incidence of reporting that we would expect for foodborne pathogens such as *Salmonella* spp. or *Campylobacter* spp. We used this output as a way of replicating how public health authorities are often made first aware of a foodborne outbreak, where an increase in incidence is noted and investigated. The parameter estimation is given in more detail in appendix B.

#### 2.3.2 Genotype generation

**Figure 4.**
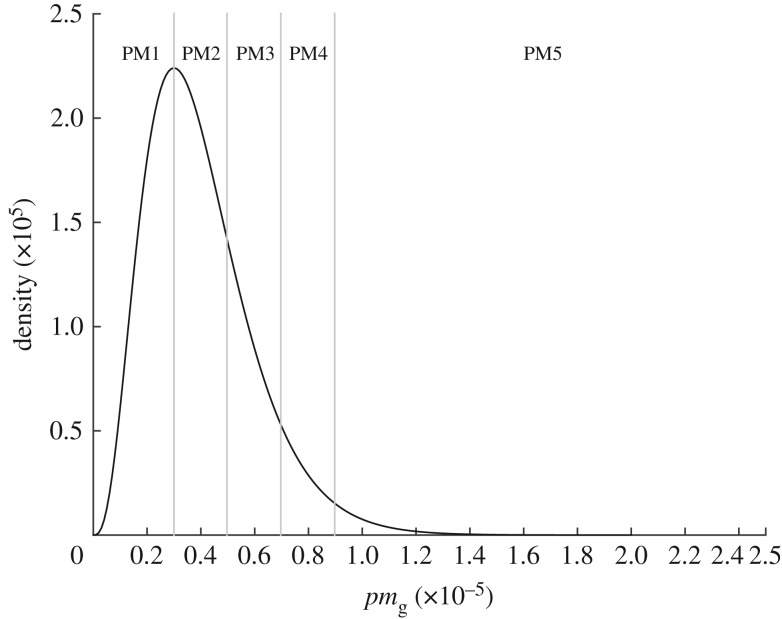
Example of how the distribution of values for the probaility of infection phenotype *pm*_*g*_ is split into five different alleles that represent the PM gene.

The *G* genotypes will have varying phenotypes with regards to survival through the processing chain or in their ability to cause human infection and initiate severe disease. The generation of phenotype from genotype is a hugely complex area, and remains one of the key challenges for being able to use NGS and WGS data within risk assessment. We therefore created a simple dummy dataset to replicate five key genes that determine the phenotypes that map to the parameters in our model: two thermal tolerance genes representing the *D*-value at 60^°^*C* and the Z-value (TTD and TTZ); attachment efficiency (ATT); dose–response (PM) and virulence (V). Each phenotype is subdivided into categories/alleles, for example, the PM gene has alleles PM1–PM5. Hence if a case sample is whole-genome sequenced, we would know if that genotype possesses the PM1 or PM5 ‘allele’. The alleles of each genotype, and their associated phenotypic responses, are listed in table 1. The distributions from which these genotype parameter estimates are taken are given in table 2: an example of the split of the phenotypic distribution into alleles is given for PM (figure 4). Each genotype was specified by its phenotypic characteristics, for example, Genotype 1 may have ATT1, TTD3, TTZ4, PM1 and V5 phenotypes.

**Table 1.** Parameter ranges for hypothetical baseline genotypes. (Each category (allele) takes its range from the associated phenotype distribution. Each genotype has the same probability of occurrence. More detail is given in appendix B.)

genotype	phenotype	description	‘alleles’	range
ATT	*β*1_*g*_, *β*2_*g*_	attachment efficiency: the proportion of strongly attached bacteria to the exterior of the product (and which will not be washed off)	A1–A5	{0,0.04,0.08,0.12,0.16,1}
TTD	*D*60_*g*_	*D*-value of genotype at 60^°^*C* on unit exterior and in water. Minutes taken to reduce bacterial subpopulation by 90%	TTD1–TTD5	{0,0.4,0.8,1.2,1.6,2}
TTZ	*Z*_*g*_	*Z*-value of genotype *g*. The temperature required for a one log (factor of 10) reduction in the *D*-value. Used with the *D*-value to calculate the inactivation rate of genotype *g* at a given temperature	TTZ1–TTZ5	{5,6,7}
PM	*pm*_*g*_	probability of human infection for one individual bacterium of genotype *g*. Used to represent the capability of human infection	PM1–PM5	{0,0.3,0.5,0.7,0.9,2}×10^−5^
V	*v*_*g*_	virulence factor representing severity of illness. We assume that the likelihood of reporting a case is directly proportional to the severity of the illness, hence *v*_*g*_ can be used to assess the under-reporting factor for genotype *g*	V1–V5	{0,0.2,0.4,0.6,0.8,1}

**Table 2.** Parameter estimates for the hypothetical food chain model.

parameter	description	distribution or equation	unit	value - mean (5th/95th percentile)	comment
*β*1_*g*_, *β*2_*g*_	proportion of firmly attached bacteria	beta(19,83)	—	0.16 (0.11; 0.22)	fitted distribution to data from [21]
*α*1_*k*_	proportion of bacteria transferring from food unit *k* to machine	uniform(0,0.0002)	—	1.0 (0.1;1.9)×10^−5^	fitted distribution to data from [21]
*N*0_*k*,*g*_	number of bacteria of genotype *g* on unit *k* at entrance to processing	lognormal(3,3)	CFU	1760 (0;2826)	assumed
*T*1_*k*_	time each unit *k* spends in stage 1 of processing	uniform(1,2)	mins	1.5 (1.05;1.95)	fitted distribution to data from [21]
*B*1_*g*_	number of bacteria of genotype *g* extruded from within interior of unit *k*	lognormal(3,3)	CFU	1760 (0;2826)	assumed
*α*2_*k*_	proportion of bacteria transferring from water to unit	uniform(0,2×10^−5^)	—	1(1.03;18.9)×10^−6^	fitted distribution to data from [21]
*D*60_*g*_	*D*-value of genotype *g* on unit at 60^°^*C*	Weibull(0.4,2)	mins	0.35 (0.09;0.70)	fitted distribution to data from [21]
*Z*_*g*_	*Z*-value of genotype *g*	uniform(5,7)	^°^*C*	6 (5.1;6.9)	fitted distribution to data from [21]
*m*	temperature decay coefficient	uniform(0,0.01)	1/*k*	0.005 (0.0005;0.0095)	assumed
*T*2_*k*_	time each unit *k* spends in stage 1 of processing	uniform(5,7)	mins	6 (5.10;6.90)	fitted distribution to data from [21]
*pm*_*g*_	probability of infection from one organism of genotype *g*	beta(4,999998)	—	4.00 (1.37;7.60)×10^−6^	assumed
*v*_*g*_	virulence/reporting factor	beta(4,8)	—	0.33 (0.13;0.56)	assumed to be around 1 in 3

#### 2.3.3 Model implementation and initial analysis of the simulated data array

The model was developed in MATLAB R2016a, using the Statistics and Machine Learning toolbox (MATLAB R2016a, The MathWorks Inc., Natick, MA, USA). We ran the model for 1000 days, with 1000 units being processed each day (so 1×10^6^ units or servings in total). We included 100 baseline genotypes, representing the broad range of phenotypes as described by the parameter estimation. The code to generate the input data and results is included in the electronic supplementary material.

The output of the hypothetical food chain model becomes the baseline dataset from which we analyse the capabilities of progressively better surveillance systems. An example of the output is given in appendix C, table 3.

**Table 3.** Example output from the hypothetical food chain model, that becomes the baseline dataset from which we draw upon to assess various surveillance systems. (The final output of the model is whether or not a food unit (serving) resulted in a reported human infection (that is *reported*={0,1}, where 1 is a reported case). Refer to notation in appendix C.)

day	unit *k*	*A*1_*k*_ (g)	*α*1_*k*_	*T*1_*k*_	*T*2_*k*_	*τ*2_*k*_	genotype *g*	*N*0_*g*_	*B*1_*g*_	*N*2_*g*_	infect	report
1	1	2	0.0001	1.3	6.3	57.8	1	1.2×10^2^	1.8×10^4^	1.2×10^3^	0	0
							2	1.5×10^2^	2.1×10^6^	1.8×10^5^	1	0
							etc.					
1	2	4.5	0.0002	1.4	6.8	57.9	1	2.4×10^2^	2.3×10^3^	1.8×10^4^	0	0
							2	2.6×10^2^	3.2×10^5^	9.8×10^3^	1	1
							etc.					
etc.												

### 2.4 Analysis progression

Owing to the preliminary nature of the study, the analyses we conducted were driven by what data we thought might realistically be collected from a food safety surveillance system (both now and in the future) rather than derived from specific policy questions. The aim of these analyses is to show the potential for, and limitations of, combining genetic and epidemiological metadata, and to inform the direction of future research. Therefore, the surveillance scenarios chosen are necessarily exploratory and dealt with at a reasonably top level. However, we still wished to describe various plausible surveillance scenarios and show where on the spectrum of data collection (from WGS data only to complete knowledge of the food chain system) we could identify the ‘signal’ of foodborne infection from the ‘noise’ of the large stochastic variation in genetic and environmental factors from food unit to food unit.

#### 2.4.1 WGS data alone 1: early detection algorithm

We started by analysing the capabilities afforded by investigating WGS data alone. Given the paucity of data available from across the food chain, this is a similar situation to where we find current food safety surveillance systems. As a surveillance tool, the only practical use of this type of isolated surveillance data is to construct time series of infectious intestinal disease, using WGS to provide greater discriminatory power to identify outbreaks. Analysis of these time series over months and years allows the construction of early detection algorithms, such as originally described by Farrington *et al.* [23]. These algorithms are used to detect anomalies in the time series of various pathogens, for example, *Salmonella* spp. or *Campylobacter* spp., that indicate whether more cases are occurring than usually expected at that time of year. Typically a moving average is adjusted for seasonality, and then confidence intervals are constructed. The upper confidence interval is used as an exceedance score: if reported cases exceed this score then a flag is raised to indicate the presence of an anomaly.

From the 1 million food units recorded in our simulated dataset, we constructed weekly totals of reported cases for each genotype *g*. As there is no seasonality in our hypothetical model (negating the need to calculate a moving average), we use the 95th percentile value of the distribution of overall weekly reported cases across all genotypes as a very basic exceedance score. This also avoids the need to choose a particular type of early detection algorithm to apply. We subsequently modified the baseline model to generate two scenarios: one where a random genotype gains 100× its previous efficiency to cause human infection, and another to investigate the effects of a systematic error in processing by introducing an average 2^°^*C* drop in stage 2 water temperature over a two-week period. We thus interrogated the time series to observe how often a flag would be raised and whether such a WGS early detection model could detect these changes from the baseline.

#### 2.4.2 Whole-genome sequencing data alone 2: whole-genome sequencing collection during microbiological sampling at processing

One of the major problems with food chain surveillance is that WGS data are only consistently collected at the human end of the food chain, with little or no characterization of WGS across the food chain. It is therefore almost impossible to identify, for example, survival genes if there is no concept of what strains did not survive the processing chain. We therefore investigated the collection of WGS data in the food chain itself.

Microbiological sampling is a legislative cornerstone of food safety within the European Union. Samples taken during processing of food (e.g. samples from pig or chicken carcasses tested for *Campylobacter* spp. or *Salmonella* spp.) allow a Food Business Operator and enforcement agencies to monitor the performance of the system to produce safe food. This microbiological sampling is legally required and hence presents a consistent and robust set of data with which to compare microbiological status of food during processing with human infection. Currently, microbiological criteria are set at species level, and so there is no requirement to assess the genomic structure of any pathogen present by NGS or WGS. However, if WGS technologies continue to become cheaper and are used on microbiological sampling isolates, we could potentially have a bounty of well-structured WGS data at the processing stage with minimal additional infrastructure and cost. Therefore, we tested the extra capability that WGS could bring by being able to link genotype at processing and human infection stages. We applied a five-samples-per-week scheme (comparable to the microbiological sampling scheme for *Salmonella* in pigs), and assumed each microbiological sample would pick up any genotype on a sample with more than 100 colony forming units (CFU) on a food unit. Therefore, over a period of 1000 days (about 143 weeks), there would be 5000 individual samples tested (we randomly pick five samples from across every week). We simply plotted the rate of reporting across genotypes against the burden of contamination by genotype.

#### 2.4.3 ‘Big’ epidemiological data: track and trace individual food units

One of the most problematic issues in food safety surveillance is being able to backtrace an outbreak along the food chain to identify the original source of the outbreak. We therefore introduced the theoretical capability to track and trace along the food chain (e.g. by RFID tagging), allowing a reported case to be allied to a sample at the stage of microbiological sampling. This would represent a significant increase in fidelity and resolution of epidemiological data, and would potentially allow us to start to look at interdependencies between epidemiological and genomic factors. We used the increasingly popular approach of machine learning to investigate whether, given sufficient data, a predictive human infection model could be developed. We do not contend that such a predictive model should (yet) be used as a decision-making tool, but we wanted to explore the potential of predictive analytics methods to strip away the noise of the food system and begin to understand the complex interdependencies between genetics and physical systems.

We assumed a perfect implementation of track and trace: that is, for each reported case we can track the food unit consumed back through the food chain and identify the processing conditions (in this case, the temperature of the hot water bath) at the time of processing that specific food unit. We also improved the assumed WGS microbiological sampling system in two ways above the previous implementation: (i) that 250 samples are taken per week to produce more cases that can be traced back to food units that were sampled during processing, and (ii) that each sample is also enumerated, so that we also have a measure of contamination burden as well as presence. Clearly, this surveillance system would be superior to anything currently available anywhere in the world, and would certainly be very expensive. However, the implementation is of interest if only for demonstration purposes, as knowing what knowledge gains such a system could bring can help us to establish where to prioritize data collection activities. For example, could simple enumeration of microbiological samples provide a bigger jump in knowledge than genomic information?

Such a surveillance system would therefore give us a dataset that identifies the processing characteristics of food units that caused reported infections, with an abattoir dataset that will tell us the genotypes on the food unit as it was processed. So for a subset of this dataset, we know which food unit caused a reported infection, the processing conditions and the genotype burdens contaminating it.

There are good, accessible reviews of machine learning in the context of genomics or epidemiology [24–26]. The basic principle of machine learning is that there are enough data to split a dataset into training and evaluation datasets. A predictive model is fitted to the training data, and then evaluated against the evaluation dataset (using some statistical measure of fit, for example, mean squared error (MSE) which we used in this study).

A very popular and relevant branch of machine learning is the use of decision trees, where the predictor variables are partitioned using a series of rules to identify regions that have the most homogeneous response to predictors. Decision trees are advantageous from a number of aspects important for food safety because they can: (i) cope with a variety of data types, (ii) the method requires no pre-processing of data, and (iii) they can deal with missing data points [24]. These are all important considerations for food chains where currently the data collected are of varying provenance and quality. The hierarchal structure of decision trees (where one input variable depends on inputs higher up in the tree) means that interactions between variables are automatically captured.

We used MATLAB's machine learning toolbox to build a predictive algorithm. A major decision when growing a predictive decision tree is to set the ‘leafiness’ or depth of the decision tree. Deep trees with many branches may very accurately reflect the training data, but are potentially over-fitted, so that the tree algorithm is not successful at prediction when applied to other (evaluation) datasets. Indeed, our preliminary analyses showed this to be the case (not shown). We therefore applied a boosted decision tree, a popular type of classification and regression tree (CART) algorithm [27] that is typically a more accurate method of producing a predictive algorithm. Boosting involves fitting a series of ‘weak learners’ (simple decision trees) to the training data. Briefly, each datapoint is first weighted equally and the first simple decision tree is fitted. The only requirement for this (and every) tree is that the prediction is better than random chance. The weights of those datapoints incorrectly classified by the first classification tree are increased so that the next decision tree fitted is focused more on fitting the incorrectly classified datapoints. This continues for as many trees as specified. A score is assigned to each classifier, and the final classifier is defined as the linear combination of the classifiers from each stage [26].

Other critical decisions when developing a boosted decision tree is the number of splits to include within each individually fitted tree (one split being equivalent to two branches within a tree). Another parameter to set is the ‘shrinkage’ learning rate, *λ*, where *λ*∈(0,1). The smaller the learning rate, the less weight given to each sequentially fitted tree; this increases the time the model takes to converge on a solution, but generally increases the accuracy of the eventual solution [25].

In our case, we have what is a relatively small, simple dataset (one response variable and six main predictor variables), but one which is characterized by a common type of problem termed class imbalance, where we have an over-abundance of non-cases and a small minority of units that lead to cases. We chose the *RUSboost* method [26], as this is designed to handle the problem of class imbalance (through random under-sampling (RUS)) as well as increasing the predictive performance of the algorithm through boosting.

Finally, we evaluated the dataset using cross-validation. Cross-validation involves partitioning a sample of the data into complementary subsets, performing the analysis on one subset (the training dataset) and validating the analysis on the other subset. To reduce variability, multiple rounds are performed using different partitions (we used five ‘k-folds’, essentially splitting the data into five roughly equal partitions). The validation results (i.e. the MSE values) are averaged over the rounds.

The following outlines the chronology of the machine learning method we implemented.

(I) Construction of the simulation dataset. We used the subsample of food units that were sampled under microbiological sampling. Based on the initial analysis of the raw dataset described in §3a, we chose to include the following variables in the machine learning dataset: (i) the contamination of the food unit at the end of stage 1 processing, (ii) the temperature of the hot water bath, and (iii) the D60, Z, PM and V alleles as our predictor variables. The binary classifier of whether a case was reported or not was the response variable.
(II) Imported this dataset into MATLAB's machine learning toolbox.
(III) We chose to implement the RUSboost decision tree method, for the reasons outlined above.
(IV) Set a range of values for the number of splits and learning rate. We investigated split number of 1,4 and 7, and a learning rate of 0.1, 0.5 and 1. We therefore generated nine predictive algorithms.
(V) We implemented five rounds (k-folds) of cross-evaluation, and used the MSE as the performance metric (the lower the MSE, the better the predictive algorithm).
(VI) We analysed the predictive capacity of the ‘optimal’ predictive algorithm (as assessed by comparing MSEs in step V).

## 3. Results

### 3.1 Simulated data array: generated by hypothetical food chain model

**Figure 5.**
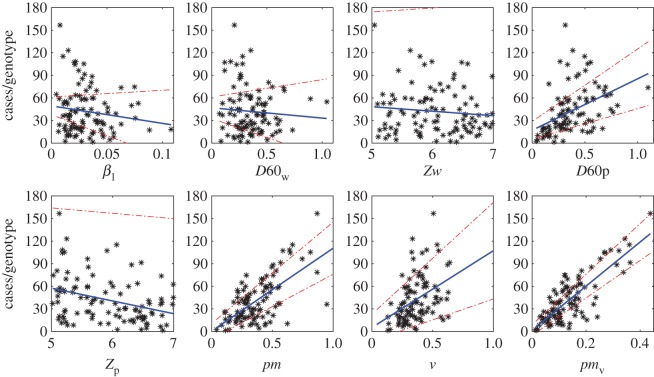
Scatterplots showing the relationships between phenotypic indicators and the number of reported cases per genotype over 1000 days. Solid blue lines represent least-squares regression fits with 95% confidence intervals (red dash-dot lines). For clarity, we show a common scale across the panels: some confidence intervals are therefore not shown (for example, the lower confidence intervals that fall below zero).

**Figure 6.**
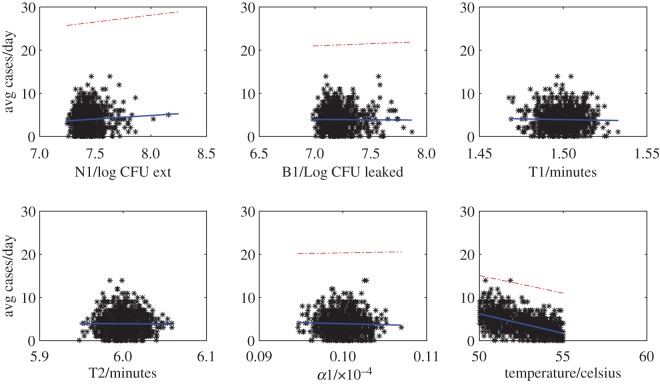
Scatterplots showing the relationships between the processing parameters (averages over day) and the average number of reported cases per day. Solid blue lines represent least-squares regression fits with 95% confidence intervals (red dashed-dotted lines). For clarity, we show a common scale across the panels: some confidence intervals are therefore not shown (for example, the lower confidence intervals that fall below zero).

The data array produced from the hypothetical food chain simulation gives, for each simulated food unit, the physical and genotype parameters and whether an infection with pathogen *g* occurred given the consumption of that food unit. This data array provides the foundation for the surveillance system analyses that follow. We first analysed the raw data array to better understand the relationships between parameters and reported infections. We plotted daily parameter averages against average number of reported cases per genotype for phenotype indicators (figure 5) and average cases per day for processing parameters (figure 6). We placed contamination rates in the processing parameter figure as these parameters are generated independently of the genotype. These simple analyses highlight that of the phenotypic indicators, only the thermal inactivation parameters while bacteria reside on the unit (*D*60_*g*_ and *Z*_*g*_) and the infection and reporting parameters (*pm*_*g*_ and *v*_*g*_), are firmly correlated with reporting rates (see appendix B for detailed definition of parameters). For the processing parameters, the temperature of the food unit appears to be a very strong driver of infection. The response to initial and post-process 1 contamination rates (*N*0_*k*,*g*_ and *N*1_*k*,*g*_) was similar and showed a modest positive correlation (only *N*1_*k*,*g*_ shown in the figure). While these simple linear fits are not robust statistical analyses (for example, in reality we would expect to see at least some nonlinear relationships, especially for those parameters driving cross-contamination), the visualizations do provide some good clues as to what parameters should drive infection and reporting rates. Therefore, we imported into the machine learning toolbox the four genotypic parameters with reasonable correlations to reported infection (*pm*_*g*_, *v*_*g*_, *D*60_*g*_ and *Z*_*g*_), plus the contamination at the end of stage 1 processing and the temperature of the processing conditions during stage 2 processing.

### 3.2 Whole-genome sequencing data alone 1: early detection algorithm

**Figure 7.**
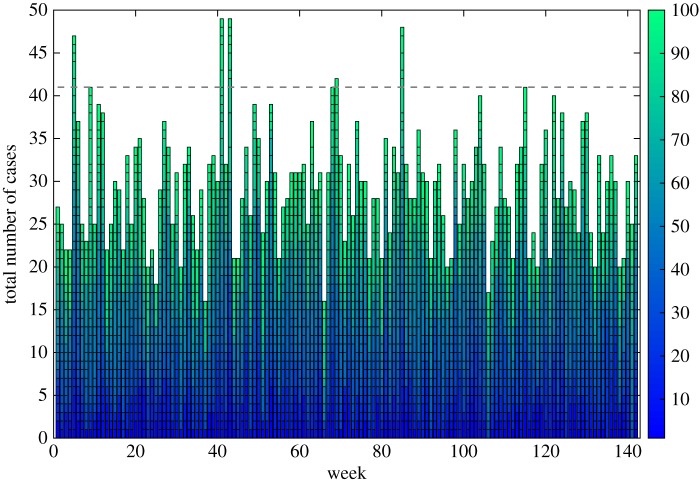
Weekly cases as generated by the hypothetical food chain model. Weekly cases by the 100 unique genotypes are stacked to produce overall numbers. The grey dotted line represents the 95th percentile, used as an arbitrary exceedance score.

The weekly totals generated from the simulated data array are displayed in figure 7. We arbitrarily set the exceedance score to the 95th percentile of total weekly cases (41 per week), such that there are roughly two to three weeks per year that exceed the exceedance score. In our baseline dataset, these flags are false positives, as there is no change from the underlying trend.

To investigate the sensitivity of this basic early detection algorithm, we modified the baseline model to mimic two types of outbreak: (i) a genotype gaining a gene or plasmid that makes it far more efficient at human infection, increasing the probability of any one organism causing infection (*pm*_*g*_) by either 10× or 100×, and (ii) a processing failure that leads to a drop in temperature of the critical control point second processing stage. We then observed how our simple early warning algorithm performs in detecting these types of changes.

**Figure 8.**
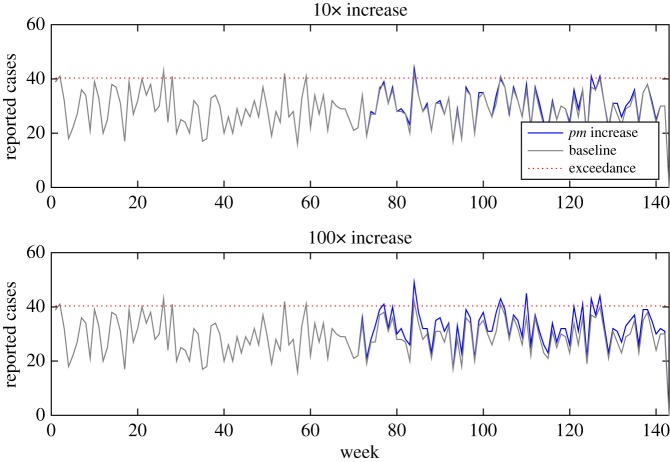
Change in overall weekly reporting if one random genotype gains efficiency to cause human infection, increasing the probability of infection from one organism by 10×–100×. Because the signal from the one genotype is swamped amongst many other genotypes, it needs a huge increase in infection efficiency (100×) to consistently breach the exceedance score.

One genotype gaining greater 10× human infection efficiency was unlikely to register within overall weekly reporting of cases; however, an increase in 100× may be notable (figure 8). For example, a genotype with 100× efficiency would make that genotype around 10–100× the efficiency of the previous maximal efficiency genotype. More detailed analysis by the responsible authorities may well detect an anomaly, even if an outbreak alarm is not triggered owing to the relatively low increase in overall cases. However, it is likely that this anomaly may be just one of many, potentially swamping any outbreak signal with noise.

**Figure 9.**
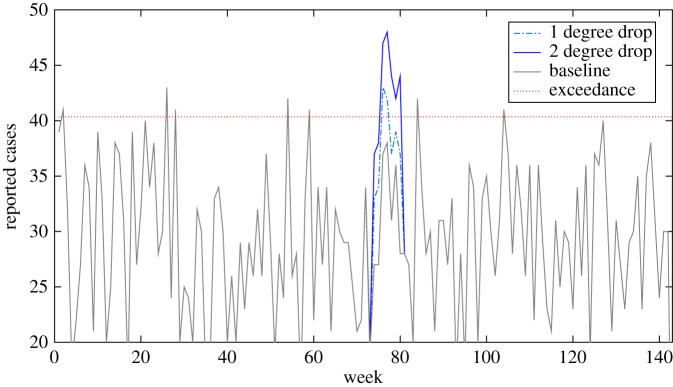
Weekly overall reported case totals: solid, baseline; dashed, average 1^°^*C* drop in processing temperature for eight weeks (weeks 73–80); dash-dot, average 2^°^*C* drop in processing temperature, same time period. Grey dotted line represents exceedance level: in the absence of more information, the 1^°^*C* drop may be missed or put down as a false positive; the 2^°^*C* would almost certainly be identified as an outbreak.

A similar pattern emerged when we temporarily dropped the average temperature of the second processing step (arbitrarily taking weeks 73–80). An average 1^°^*C* drop in temperature led to a clear increase in cases (figure 9), but may well be mistaken for a false flagged event if further information (e.g. follow-up questionnaires for reported cases) were not sensitive enough to identify the processing establishment as the common link. A 2^°^*C* drop led to a bigger, more sustained exceedance of the threshold, and would therefore increase the likelihood of detecting the processing failure. Both temperature failure scenarios could therefore be detected by an early warning system, but it may require some significant detective work in the 1^°^*C* scenario. An average of 1–2^°^*C* is a large drop in scalding temperature, and it is likely such drops in temperature would start to affect the quality of the food product. Hence, the processing establishment may well detect and rectify the problem themselves much sooner than the surveillance system could.

### 3.3 Whole-genome sequencing data alone 2: the addition of whole-genome sequencing to microbiological sampling criteria

**Figure 10.**
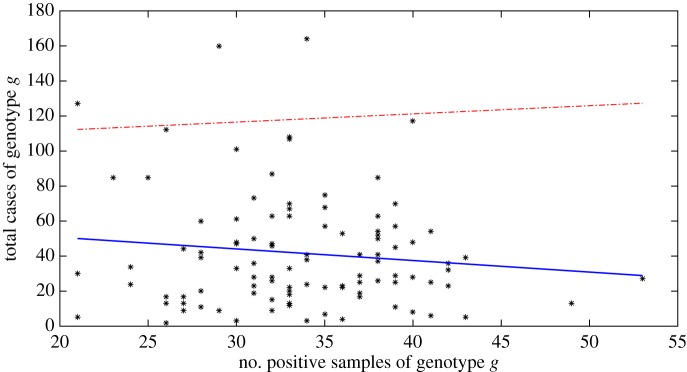
Scatter plot of the total number of genotype *g* samples isolated during process microbiological sampling over 143 weeks of processing (five samples every week). The solid blue line is the least-squares regression fit, the red dash-dot line the upper 95% confidence interval. We have removed the lower confidence interval for clarity.

We plotted the number of genotype *g* isolated during microbiological sampling over the 143 weeks against the number of human cases attributed to genotype *g* (figure 10). The scatterplot clearly shows the weak relationship between WGS information at the abattoir and reported cases; the wide regression confidence interval supports this conclusion. We are clearly missing some crucial information. This result is not unexpected. Even increasing the number of samples taken per week to 250 did not show any relationship between abattoir and reported infection (not shown).

### 3.4 ‘Big’ epidemiological data: track and trace individual food units

**Figure 11.**
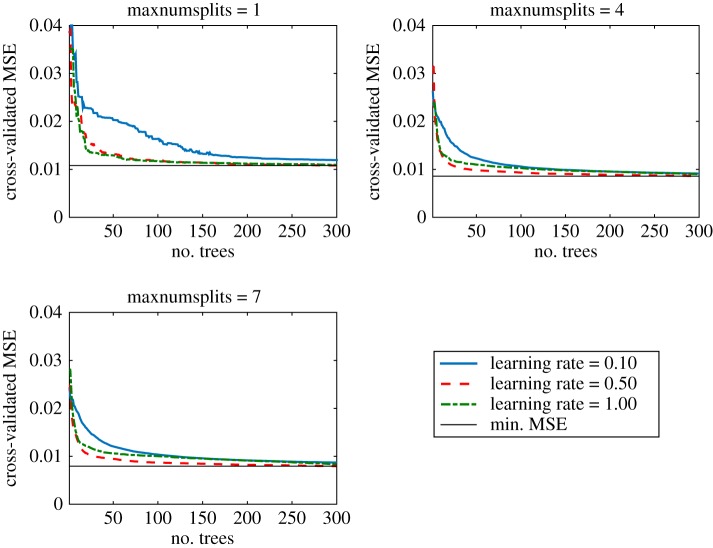
Comparison of the performance of the *RUSBoost* algorithm in predicting the reporting of human cases for varying number of splits in the trees (maxnumsplits), the number of fitted trees in each algorithm (*x*-axis) and for learning rates 0.01–1. We used the cross-validated mean squared error (MSE) as the measure of performance. Using seven splits produced the best-performing algorithm; there was little performance increase using more than 200 trees per algorithm.

Using the MSE as described in step 5 of the machine learning method, the optimal predictive model was one with a maximum of seven splits per tree, and a learning rate, *λ*, of 0.5 (figure 11). There is a slight increase in performance between three and seven splits, but little was added to the performance by increasing splits above seven (not shown). We chose to use 300 decision trees, but there was little improvement in the MSE above 200 trees.

**Figure 12.**
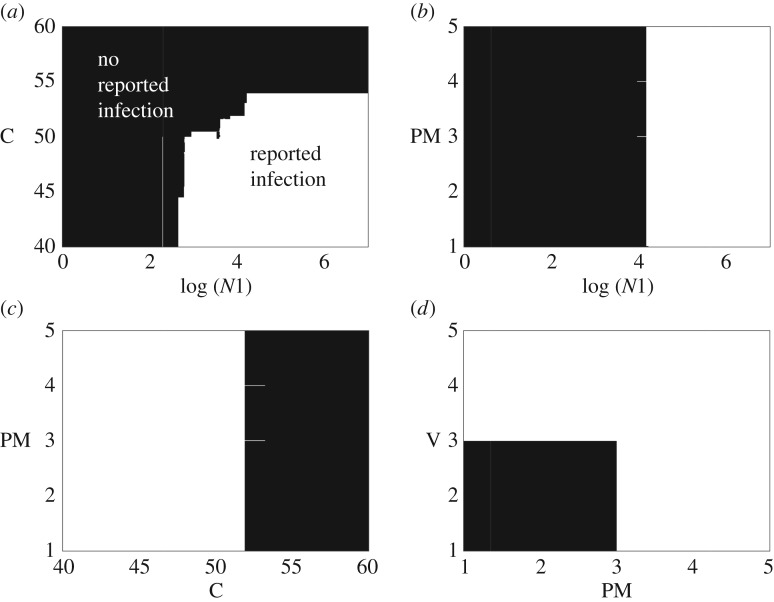
Decision surfaces for selected comparisons. Those combinations of parameters classified as no reported infection are coloured black, those classified as reported infection are white. Clockwise from (*a*) log contamination of exterior at the end of stage 1 processing (*N*1) versus temperature of hot water bath (C); *N*1 versus the allele associated with probability of infection, PM; C versus PM; and finally PM versus the virulence allele, V. When not included in the comparison, the following parameters were set at the median values: *PM*=2, *V*=2, *D*60=1, *Z*=1. We set *N*1=1.4×10^4^ CFU and *C*=52.8^°^*C* to produce boundary conditions that allowed us to show the impact of the PM and V alleles in (*a*,*c*,*d*) panels.

Most trees are dominated by nodes splitting the level of contamination at the end of stage 1 processing (*N*1_*k*,*g*_), followed by the temperature of the stage 2 hot water bath (*C*_*k*_) (not shown). Of the genotypic factors, the PM allele is the most common, but appears relatively infrequently compared to the physical parameters. This strongly suggests that contamination burden (regardless of genotype) is a key influence of whether or not infection is reported. Indeed, the impact of contamination level and temperature is clear in the decision surface produced by using the predictive model to predict classification of reported infections for various contamination levels and temperatures (figure 12*a*). As we would expect, combinations of higher contamination and lower temperatures drive the decision surface classified as reported infection. Figure 12*b*,*c* illustrates the importance of ‘physical’ parameters (contamination level and temperature) relative to the most influential genotypic factor, the probability of infection. The PM allele makes little difference to the classification of a unit, except when right on the cusp of the decision boundary between contamination level and temperature. For example, only fitter PM and V alleles are classified as reported infections when on the decision boundary of contamination level and temperature (using the decision boundary that occurs at 1.4×10^4^ CFU and 52.8^°^*C*; figure 12*d*). Thus, we could hypothesize that there is a large decision space where only classic parameters such as contamination level and critical control points such as scalding water temperature are important. Genotypic alleles are therefore only important on the cusp of decision boundaries. Given food hygiene processes such as hazard analysis and critical control points (HACCP), these decision surface boundaries are hopefully most commonly approached when there is a failure in the food production chain.

**Figure 13.**
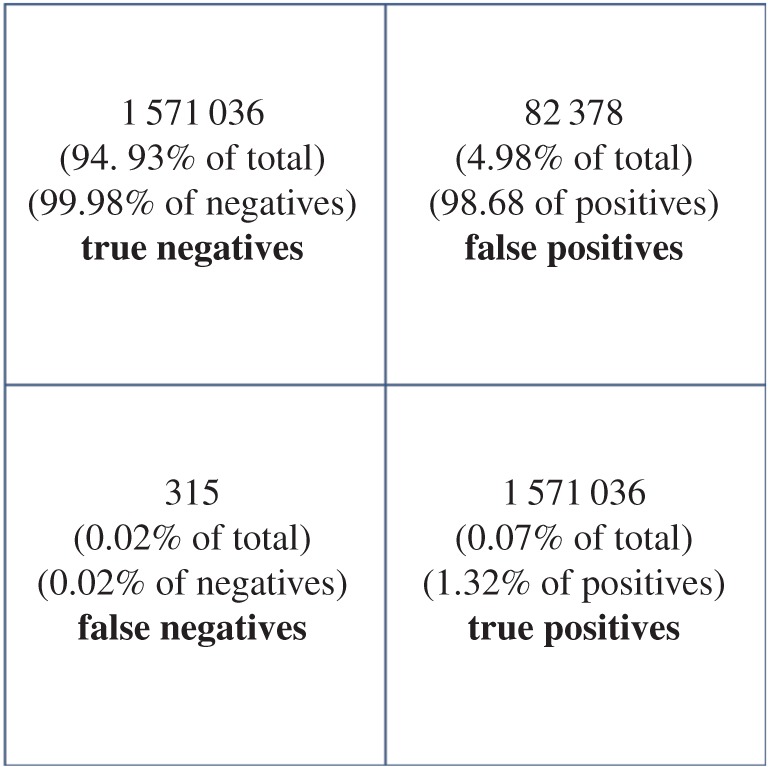
Confusion matrix for the best performing model (max number of splits, 7; learning rate 0.01; number of trees: 200).

One of the key aspects of machine learning is the division of the original dataset into training and evaluation datasets. The training dataset is used to develop the boosted decision tree solution, and the evaluation dataset is used to test the predictive power of the predictive algorithm. We therefore assessed the performance of the optimal predictive algorithm to predict whether a case is reported using the evaluation dataset set aside from the original dataset. This information is displayed using a confusion matrix (figure 13), which describes the number of false positives, false negatives, etc. From these figures, we can work out the sensitivity of the predictive model, that is *Se*=1102/(1102+315)=0.78, and the specificity, *Sp*=1 571 036/(1 571 036+82 378)=0.95. Therefore, our predictive model would be relatively good at detecting combinations of environmental and genomic characteristics that lead to cases. Hence, if such big data were available, the authorities could very well use such techniques to identify and monitor potentially high-risk scenarios. However, our results exhibit a classic example of the problem of looking for a rare event: we can detect roughly three out of four cases, but the total predicted cases are dominated by false positives owing to an over-abundance of negative results. Therefore, the predictive power of the model is poor: the positive predictive value is only 0.01.

## 4. Discussion

Our hypothesis stated that to generate genuine knowledge from high-fidelity genomic data for food safety surveillance we should also link it to *relevant*, high-fidelity epidemiological metadata, using data from across the food chain. Our analyses therefore focused on identifying where a useful step-change in knowledge may be generated from ever more detailed genomic or epidemiological metadata. In short, our preliminary conclusions are that tracking and tracing food units at as high a resolution as possible is necessary to genuinely transform (genetic) data into knowledge of epidemiological risk. However, the majority of extra knowledge gained from tracking and tracing was because of being able to determine the success of the food chain in controlling contamination burden, regardless of the genetic data associated with a case or food unit. Only on the cusp of decision boundaries (that is where small changes in either contamination or the efficiency of the food chain to mitigate that contamination tip the balance in favour of infection or no infection) did we see the influence of genetic factors.

We first tested a surveillance scenario that looks much like what is present in the UK now: using genotyping information from reported cases to interrogate weekly cases trends. We showed that changes in the baseline system have to be quite large to be detected—a consequence of the huge variation that exists in a food production system (even from our simple food chain model). This means that the exceedance score has to be set reasonably high to avoid a burdensome false positive rate, otherwise authorities would expend precious resources on following up what are in all likelihood spikes in the natural trend rather than genuine outbreaks. Without further contextual information, the resolution of the prediction being made of these early warning systems is relatively low. It simply states ‘there may be an outbreak’, but potentially significant epidemiological investigations may be needed to establish if there is an outbreak, and if so what is the origin. These algorithms (of themselves) do not answer the questions ‘why did it happen?’ or ‘what will happen?’

Currently, WGS has shown its value in some outbreaks where the same genotype can be linked to a single source and may well accelerate source identification or provide robust evidence for one particular contamination source over others [12,28,29]. However, the main purpose of outbreak investigation is to control and mitigate the source of infection; while some useful insights may be gained by these investigations, they do not provide a standardized and robust way to link genomic and epidemiological data, and indeed the epidemiological data collected can be inconsistent and incomplete owing to the nature of backtracing along a complex and dynamic system.

Our second surveillance system included the data from the first scenario (reported case WGS data), plus WGS information from the processing stage. This would be an advance over most current surveillance systems across Europe, but is not that unrealistic, as we assumed we could access samples from mandated microbiological sampling. However, there was no clear correlation between rates of WGS at processing and reported cases (figure 10). This is not unexpected, given that such a system would be based on the presence of a genotype, rather than burden. Contamination levels (in this model, and observed in many field studies of food and animal samples) regularly vary across many orders of magnitude: if contamination level is not enumerated, this wide variance is very likely to overwhelm any signal with noise.

Of wider interest is that implementing WGS in microbiological sampling schemes produces data that could be used within the common and popular source attribution models based on the seminal work by Hald *et al.* [30]. Their model is based on the principle of being able to construct joint distributions for a couple of key parameters that describe the food-dependent and bacteria-dependent factors. However, one of the major issues with this type of Bayesian model is the sensitivity of the final joint probability distribution to the choice of prior information, and commonly the lack of enumeration data as mentioned above. This is critical as surveillance systems rarely generate the data to provide prior distributions for these parameters (especially contamination burdens) because food surveillance systems do not collect data in sufficient detail from across the food chain. While the Hald models provide valuable information for source attribution and the prioritization of resources to the most problematic sources, our hypothetical analysis shows that better contextual data from across the food chain, especially on contamination burden, is still necessary, especially if these methods are to be used with WGS information (either to better populate the Hald models or to use new approaches). Similar conclusions were reached by the authors of a Danish study investigating the use of multiple locus variable-number tandem repeat analysis (MLVA) methods for *Salmonella* attribution [31,32].

The most important aspect that increased the fidelity of metadata was to add the ability to track and trace individual units. Without this detailed track and trace data, the noise of even our simple food chain overwhelmed any patterns that we may have been able to identify. The machine learning example was able to identify numerous interactions that lead to cases, of which most rely simply on the contamination level at the end of stage 1 processing and the temperature of stage 2 processing (figure 12). The contribution of genetic information was limited in comparison, although the *RUSBoost* algorithm did identify that more efficient infection potential (PM3–PM5 alleles) and higher virulence (V3–V5) were associated with cases on the decision surface boundary between contamination level and temperature (figure 12). Thus, given appropriate surveillance data, machine learning does provide a potentially attractive option for analysing food chain data, with the ability to determine the importance of interactions between novel genes/alleles and the processing environment. A further research project could concentrate on an area where we have real, relatively good cross-food chain data, for example, a big integrated supply chain.

The predictive model was reasonably sensitive if all information was known about a food unit, but the predictive value of the model is limited owing to the rare nature of foodborne infection. In our hypothetical model, there were around a thousand units consumed for every reported case. Thus, even though the predictive model is quite specific (95% specificity), the number of false positives dwarfs the number of true positives. This is the well-known issue of class imbalance and occurs in any surveillance system dealing with relatively few numbers of positives. Hence, we do not suggest that such a predictive model is currently used to ‘predict’ outbreaks or the burden of foodborne illnesses, but rather is used to identify scenarios of combined environmental and genomic characteristics that may prescribe a higher risk of infection in the consumer population. This could supplement current early detection algorithms, either to improve the predictive power of such algorithms, or to provide extra context to qualitative horizon scanning efforts, for example, to assist in the identification of the provenance of an outbreak.

There are of course large barriers to the implementation of a system that can track and trace food units or batches. A survey of poultry and pig producers conducted during the study showed that the ability to track and trace food products is varied. Some companies do have centrally collated electronic databases of sampling results and processing data, but some only have locally stored paper records, which may or may not be interrogated further. There would also probably need to be investment in the technologies required to transmit, store and collate the large volumes of data generated by real-time sensors. Hence, the introduction of RFID tagging would have to be shown to be cost-effective. Even if RFID tracking and tracing was introduced, under current arrangements this information would not be immediately available to local or national authorities. Hence, there are several issues around data generation and access that would have to be surmounted first. One attractive area of research would be to establish exactly what fidelity of track and trace data may both improve food safety surveillance but also production performance. If these RFID technologies are shown to be beneficial in improving production efficiency, then food chain companies may well take up the technologies for their own purposes, with food safety surveillance being improved as a byproduct.

Big data approaches are gaining in popularity, and are hailed as a way to improve decision-making and increase competitiveness in the market [33]. They have been applied in a variety of related fields in genetics (genomics and proteomics), but detailed metadata are rarely taken into account, and as such methods tend to focus exclusively on the information that can be gained from the genetic data alone (presumably because there is usually a lack of complementary metadata). However, as we have shown through both classical statistical and big data approaches, we should not ignore epidemiological and contextual data. The burden of contamination and the processing environment, rather than the genetics of a novel strain, are still the most fundamental aspects for control of foodborne pathogens. A novel, virulent foodborne pathogen cannot cause illness if exposure does not occur, or doses ingested are too small to cause infection. As always, we must consider both the opportunity for exposure and the capability of infection/virulence of disease.

The advantage of big data is that, given relevant data sources, we should be able to collect enough data to fully represent the true distribution of variables and outputs, something that we cannot be confident about current data availability. This in itself would be a big step change in the field of food safety, where analysts commonly have to work with poor, incomplete data with small sample sizes. Paying significant attention to data generation, curation and analysis through the application of logistical and technological advances to both genomic and supply chain data would undoubtedly improve the representativeness of collected data compared to the current situation. This improvement in data curation and collection is a first priority above and beyond attempting to build models that can predict outbreaks or foodborne illness.

The research presented here is preliminary and theoretical, but directed at the question of how big metadata would support the development of more sensitive surveillance systems based on WGS technologies. The surveillance scenarios, and their analyses, are necessarily exploratory and presented at a top level. Detailed study of the application of big data to specific food surveillance policy questions will come later, but hopefully this research will inform the direction of that research. A lot of research and development is required between now and the application of the machine learning methods to directly influence operational activities and epidemiological investigations. This includes: ongoing research into translating genomic data into risk-based knowledge of genotype through matching the most relevant, high-fidelity epidemiological data to genomic data; researching other machine learning methods to develop and improve the predictive/insight capability of such methods; liaising with industry to determine the reception of companies to employ (and share the results of) big data systems; and to conduct operational research activities to identify the optimum real-time data gathering processes that allow industry to gain better productivity and that allows both industry and government to identify the most cost-effective measures for food safety.

## Supplementary Material

MATLAB .m file: Code for food chain model that generates simulated baseline data array. Surveillance scenario analysis code also included. No other data or code are required to replicate results

## Data accessibility

All data used in the study was hypothetical and generated via the simulation model. The code to generate the model and the data input for the machine learning model is attached as the electronic supplementary material.

## Appendix A. Mathematical description of the food chain model

The model is derived from Swart *et al.* [21]. We add a subscript *g* to the original equation parameters to incorporate different genotypes (with varying abilities to survive the food chain and cause infection). For simplicity, we assume the individual populations of genotypes act independently from each other.

**A.1. Cross-contamination process (unit → equipment → other units)**

The important aspect of this process is the extrusion of contaminated material from unit *k* to the equipment, which then cross-contaminates other units with pathogen *X*_*g*_ (akin to evisceration or dehairing). Using coupled recursion equations, we can calculate the number of pathogen *X*_*g*_ on the equipment during processing of unit *k*, $W1_{k,g}^{-}$, and on the unit *k* after processing, *N*1_*k*,*g*_ as follows:

A 1*a* $$W1_{k+1,g}^{-}=[\beta 1_{g}(N0_{k+1,g}+B1_{k,g})+W1_{k,g}^{-}]e^{-\alpha 1_{k}T1_{k}}$$

and

A 1*b* $$N1_{k,g}=W1_{k,g}^{-}(e^{-\alpha 1_{k}T1_{k}}-1)+(1-\beta 1_{g})(N1_{k,g}+B1_{k,g}),$$

where *N*0_*k*,*g*_ and *B*1_*k*,*g*_ are the initial contaminations of unit *k* of genotype *g* in CFUs on the exterior and interior, respectively. *β*1_*g*_ is the proportion of firmly attached bacteria for genotype *g* that we assume will remain on unit *k* and *α*1_*k*_ is the fraction of the free bacteria that transfer from the unit *k* to the evisceration machine. The unit spends *T*1_*k*_ minutes in the evisceration/dehairing machine. We assume *W*1^−^ at *k*=0 is zero.

**A.2. Thermal inactivation process in hot water bath**

Assuming a line of units is submerged in a hot water bath after the first process, the solutions for the number of pathogen *X*_*g*_ residing in the hot water bath or on the exterior of unit *k* ($W2_{k+1,g}^{-}$ and *N*2_*k*+1,*g*_) at the time of processing are:

A 2*a* $$W2_{k+1,g}^{-}=[\beta 2_{g}N1_{k,g}+W2_{k,g}^{-}]e^{-\gamma 2_{g}(1-\alpha 2_{k})T2_{k}},$$

and

A 2*b* $$N2_{k+1,g}=W2_{k+1,g}^{-}\left[\frac{\alpha 2_{k}}{\gamma 2_{g}(1-\alpha 2_{k})-\gamma 2_{k}}\right](e^{[\gamma 2_{k,g}(1-\alpha 2_{k})-\gamma 2_{k}]T2_{k}}-1)+(1-\beta 2)N2_{k,g}e^{-\gamma 2_{k}T2_{k}},$$

where *α*2_*k*_ and *β*2_*k*_ have the same definitions as their counterparts for stage 1 processing. The parameter *γ*2_*g*_ is the inactivation of genotype *g* at temperature *C*_*k*_. We assume *W*2^−^ at *k*=0 is zero. The inactivation coefficient *γ*2_*k*_ is calculated by

A 3 $$\gamma 2_{k}=\frac{1}{D60_{g}}\times 10^{(C_{k}-60)/Z_{g}},$$

where the *D*-value parametrization is given for 60^°^*C*.

We assume that as processing through the day continues, the temperature of the hot water bath decreases over time from a baseline of 55^°^*C* as more material is mixed into the water. We also assume a cyclical nature to the temperature over time, as operators monitor and adjust temperature back upwards. Therefore, the temperature each unit *k* is processed at is given by

A 4 $$C_{k}=55-mk+\left(5\times \sin\left(\frac{\pi }{4}k\right)\right),$$

where *m* is the gradient coefficient of temperature decay.

**A.3. Extension to case reporting**

For simplicity, we assume that there is no further cross-contamination, growth or inactivation along the food chain, and that a person consumes the whole of unit *k* in one meal. Therefore, the dose ingested by a person during one meal made with one of our processed units is the same as the number of bacteria within each population *g* at the end of stage 2 processing, that is, *D*_*k*,*g*_=*N*2_*k*,*g*_.

In the absence of any other data, we make the same simplifying assumption for the dose–response model as we did for the inclusion of separate genotypes above, that all sub-populations of *X* act independently. Therefore, the probability of infection of a single person is given by

A 5 $$P_{k,g}∼\sum [1-{\left(1-pm_{g}\right)}^{D_{k,g}}].$$

The likelihood of reporting an infection as a laboratory-confirmed case is assumed proportional to a virulence parameter *v*_*g*_. Therefore, the probability of a reported case is

A 6 $$L_{k,g}=P_{k,g}v_{g}.$$

## Appendix B. Parameter estimation

The parameter estimates for the hypothetical food chain model are shown in table 2. Where possible, we generate the variation in the baseline parameter estimates from Swart *et al.* [21] (we note where this is not the case). The parameter estimates from this paper are specific to *Salmonella* spp. in pigs, but we use them simply as a way to generate some realistic values for processing parameters and a classical bacterial thermal tolerance or infection potential. We assume the variation in the parameter estimates represent the phenotypic variation across a species, derived from the specific genetic characteristics of each genotype.

## Appendix C. Simulated output

The food chain model is ran for 1000 iterations to represent 1000 days of processing (where 1000 units per day are processed). Table 3 displays an example of the output from the hypothetical food chain model (numbers do not necessarily reflect the final output used in the analysis).
